# Complete Genome Sequence of Phascolarctobacterium faecium G 104, Isolated from the Stools of a Healthy Lean Donor

**DOI:** 10.1128/MRA.01054-20

**Published:** 2021-01-28

**Authors:** Sadjia Rachek, Naghmeh Nikpoor, Eva M. Gómez del Pulgar, Aitor Gonzaga, Yolanda Sanz, Thomas A. Tompkins

**Affiliations:** a Rosell Institute for Microbiome and Probiotics, Montreal, Quebec, Canada; b Microbial Ecology, Nutrition, and Health Research Unit, Institute of Agrochemistry and Food Technology, Spanish National Research Council (IATA-CSIC), Valencia, Spain; University of Maryland School of Medicine

## Abstract

Phascolarctobacterium faecium is a strict anaerobe belonging to the *Firmicutes* phylum that is found abundantly in the human gastrointestinal tract. Here, we report the complete genome sequence of P. faecium G 104, a strain isolated from a fresh stool sample from a healthy lean donor.

## ANNOUNCEMENT

Phascolarctobacterium faecium is a Gram-negative, nonsporulating, and strictly anaerobic bacterium that was first isolated from a healthy human donor (1) and was characterized as a succinate-utilizing bacterium and substantial acetate/propionate producer (2). This bacterium was reported to positively correlate with maintenance of normal weight in children (3) and positive mood in humans (4) and to lower levels of liver triglycerides following a high-fat diet in a nonalcoholic fatty liver rat model (5). P. faecium strain G 104 was isolated from the healthy donor’s fecal sample, using intestinal bacteria medium (IBM) (6) supplemented with different carbohydrates (0.2% xylan, 0.2% wheat bran extract, 0.1% arabinogalactans, 0.1% Arabic gum, 0.5% starch, and 0.1% inulin). Feces were inoculated (1:5) in 50 ml of IBM and incubated for 24 h in an anaerobic chamber (Whitley DG250 workstation) with stirring and pH control (6.9 to 7.0). Fermented IBM was filtered and added as a supplement to fastidious anaerobe agar (FAA) supplemented with 0.5% defibrinated sheep blood. Serial dilutions of feces previously incubated in IBM were plated on FAA and incubated under anaerobic conditions at 37°C for 72 h. Colonies were isolated, including P. faecium strain G 104. The strain was purified by streaking a single colony onto peptone yeast extract agar supplemented with 50 mM sodium succinate. Genomic DNA was isolated by the phenol-chloroform extraction method. A DNA library was prepared according to the Pacific Biosciences protocol for preparing multiplexed microbial libraries using the SMRTbell Express template preparation kit v.2.0 and was sequenced on the PacBio Sequel II system at the Genome Quebec facility (Montreal, Canada). The single-molecule real-time (SMRT) Link software v.6.0.0 was used to filter and assemble the subreads. The HGAP4 algorithm (7) was used to correct and preassemble the filtered subreads, which were then assembled into contigs. All reads were polished and processed by alignment with contigs using Arrow. Circlator v.1.4.1 (8) (https://github.com/sanger-pathogens/circlator) was used for circularization and removal of the overlaps, as described in the PacBio assembly sequencing pipeline (9). Contigs were analyzed using BLAST+ v.2.3.0. Default parameters were used for all software unless otherwise specified. A total of 44,197 filtered longest subreads were obtained, with an *N*_50_ value of 6,395 bp, filtered subread coverage of 77,882×, and a base count of 233,646,961 bp. The genome was rotated so that its start coincided with that of strain JCM 30894 (10). The final assembly resulted in a single circular contig. Genome annotation was performed using the NCBI Prokaryotic Genome Annotation Pipeline (PGAP) (11). The genome consists of a single 2,364,938-bp circular chromosome, with a GC content of 43.8%. Genome annotation predicted a total of 2,256 genes, 2,183 genes with coding sequences, and 2,125 protein-coding genes, including 73 RNA-encoding genes (54 tRNAs, 15 rRNAs, and 4 noncoding RNAs), with 58 pseudogenes. Investigation of a putative annotated genome revealed a complete pathway for biosynthesis of vitamin B_12_. Fifteen genes involved in lactate fermentation, butanol biosynthesis, and acetyl-coenzyme A (CoA) fermentation into butyrate and three genes encoding a bile acid sodium symporter and bile acid-inducible protein F were detected but not functionally determined.

### Data availability.

The complete genome sequence of P. faecium G 104 was deposited in GenBank under accession number CP061002.1, BioProject accession number PRJNA659610, BioSample accession number SAMN15921107, and SRA accession number SRR13005647.
